# Supplementary material for: Human Adenovirus Type 5 Infection Leads to Nuclear Envelope Destabilization and Membrane Permeability Independently of Adenovirus Death Protein
Source: Int J Mol Sci. 2021 Dec 2;22(23):13034. doi: 10.3390/ijms222313034 (PMC8657697; doi:10.3390/ijms222313034)
Supplement: Supplementary file 1 [file ijms-22-13034-s001.zip › Pfitzner_et_al_2021_supplementary-materials.pdf]

Supplementary materials

Graphical Abstract: Artistic representation of nuclear egress by HAdV5. This figure was based on Figure 6c from the main text. For clearer interpretation of the virus particles, the figure was modified by adding four 3D capsid images. The used images are different views of a filtered, single particle analysis HAdV5 electron microscopy map (Franken *et al.*, unpublished).

Movie S1: 3D visualization of the membrane damage site of HAdV5 pIX-mCherry ADP+. An example of nuclear envelope damage detected in infection with HAdV5 pIX-mCherry ADP+ is shown. For this movie, A549 cells were infected and prepared for serial block-face SEM at 42 hpi. Sequential z-slices were combined to a 3D image using Imaris (Bitplane).

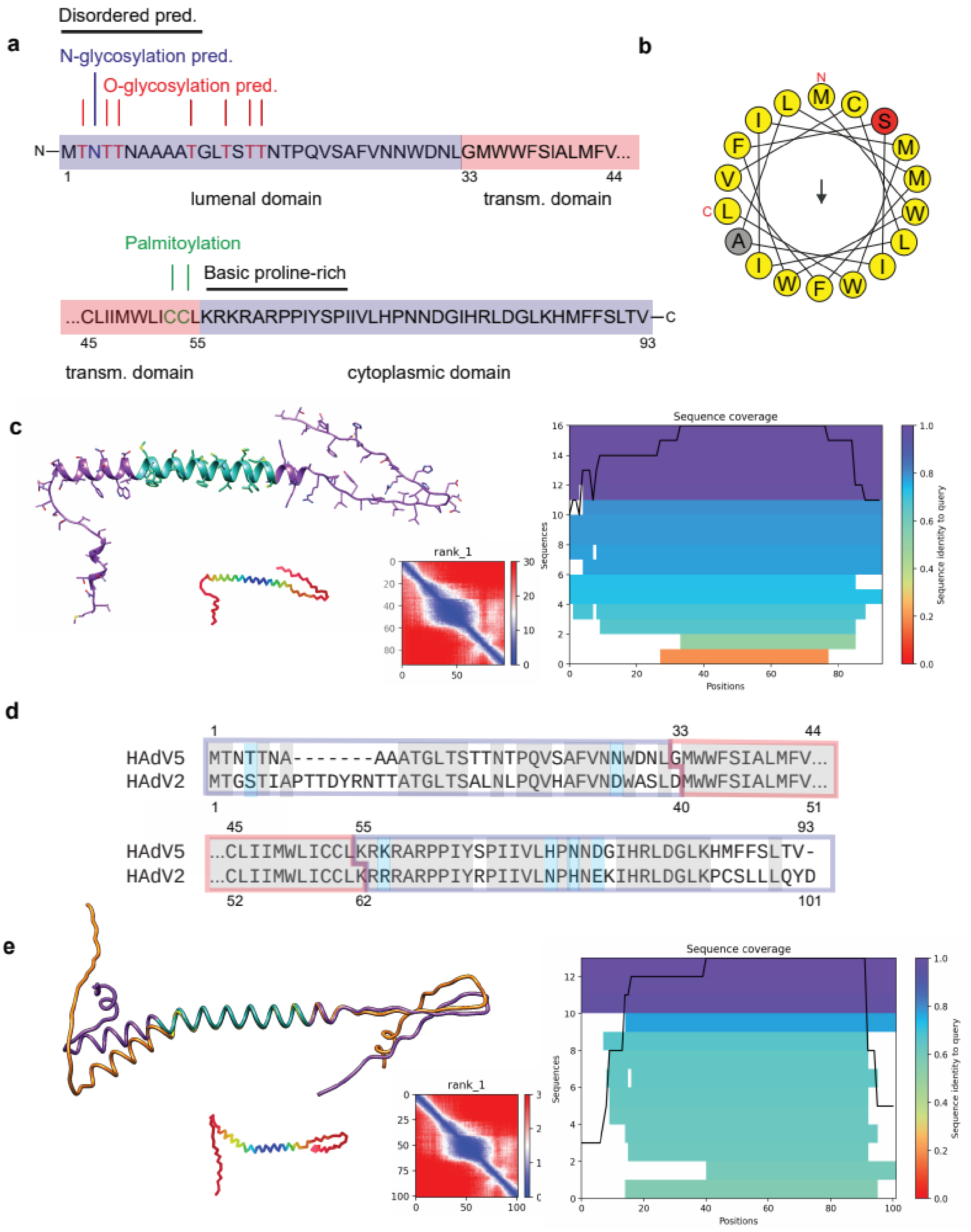

Figure S1: Bioinformatic analysis of structure and function of HAdV5 ADP and comparison with HAdV2 ADP. (**a**) Overview of the HAdV5 ADP protein sequence including confirmed and putative PTMs and domains. ADP is a short protein of 93 amino acids consisting of a single-spanning transmembrane domain. A basic region in the cytoplasmic domain could act as nuclear localization signal and the N-terminal region of ADP is predicted to possess intrinsically disordered properties. Single PTMs are

predicted to consist of N-glycosylation at residue Asn3, O-glycosylation at residues Thr2, 4, 5, 11, 14, 16 and 17 and palmitoylation at residues Cys53 and Cys54. Structural and PTM predictions were run using GPS-Lipid [1], JPred [2], NetOGlyc [3], PrDOS [4] and SPRINT-Gly [5]. (**b**) The ADP transmembrane helix has a hydrophobic surface and does not possess amphipathic properties. Small amino acids (Gly, Ala) are colored in grey, hydrophobic amino acids are colored in yellow and hydrophilic amino acids are colored in red. The transmembrane helix analysis was performed using Heliquest [6]. (**c**) Structure prediction of HAdV5-ADP by AlphaFold2 [7] confirms lack of amphipathic properties and presence of transmembrane domain. The small ADP icon is colored based on the AlphaFold2 pLDDT (red least confident, blue very confident), the table labeled rank 1 gives the predicted alignment error (red least confident, blue very confident) and the right table shows the sequence coverage on which the prediction is based. Both Phyre2 [8] and TMHMM [9,10] confirm the predicted transmembrane helix between amino acids 33 and 54 or 55 respectively. This domain is shown in green on the AlphaFold2 structure. (**d**) A sequence comparison between ADP of HAdV5 and HAdV2 was performed with ClustalW [11], EMBOSS Needle [12] and Blastp [13,14], which were all in agreement. While ClustalW and EMBOSS Needle gave an overall sequence identity of 67 out of 101 amino acids (66.3%) and a similarity of 74 out of 101 amino acids. Blastp took only amino acids 10-85 from HAdV5-ADP and 17-92 from HAdV2-ADP, which lead into an 82% sequence identity. (**e**) Comparison of the Alphafold2 structure prediction of HAdV5 ADP (purple and seagreen) and HAdV2 ADP (orange and yellow). The small icon depicts HAdV2-ADP and is colored based on the AlphaFold2 pLDDT (red least confident, blue very confident), the table labeled rank 1 gives the predicted alignment error (red least confident, blue very confident) for HAdV2-ADP and the right table shows the sequence coverage on which the prediction of HAdV2-ADP is based. Both Phyre2 [8] and TMHMM [9,10] confirm the presence of the same transmembrane helix in HAdV2-ADP, but now it is located between amino acids 41 and 61 or 63 respectively. This domain is shown in yellow on the AlphaFold2 structure and is fully conserved. Structure predictions were visualized using UCSF Chimera [15], developed by the Resource for Biocomputing, Visualization, and Informatics at the University of California, San Francisco, with support from NIH P41-GM103311. While the Alphafold2 predictions are not confident, especially for the predicted disordered domains of the proteins, they hind towards potential structural differences.

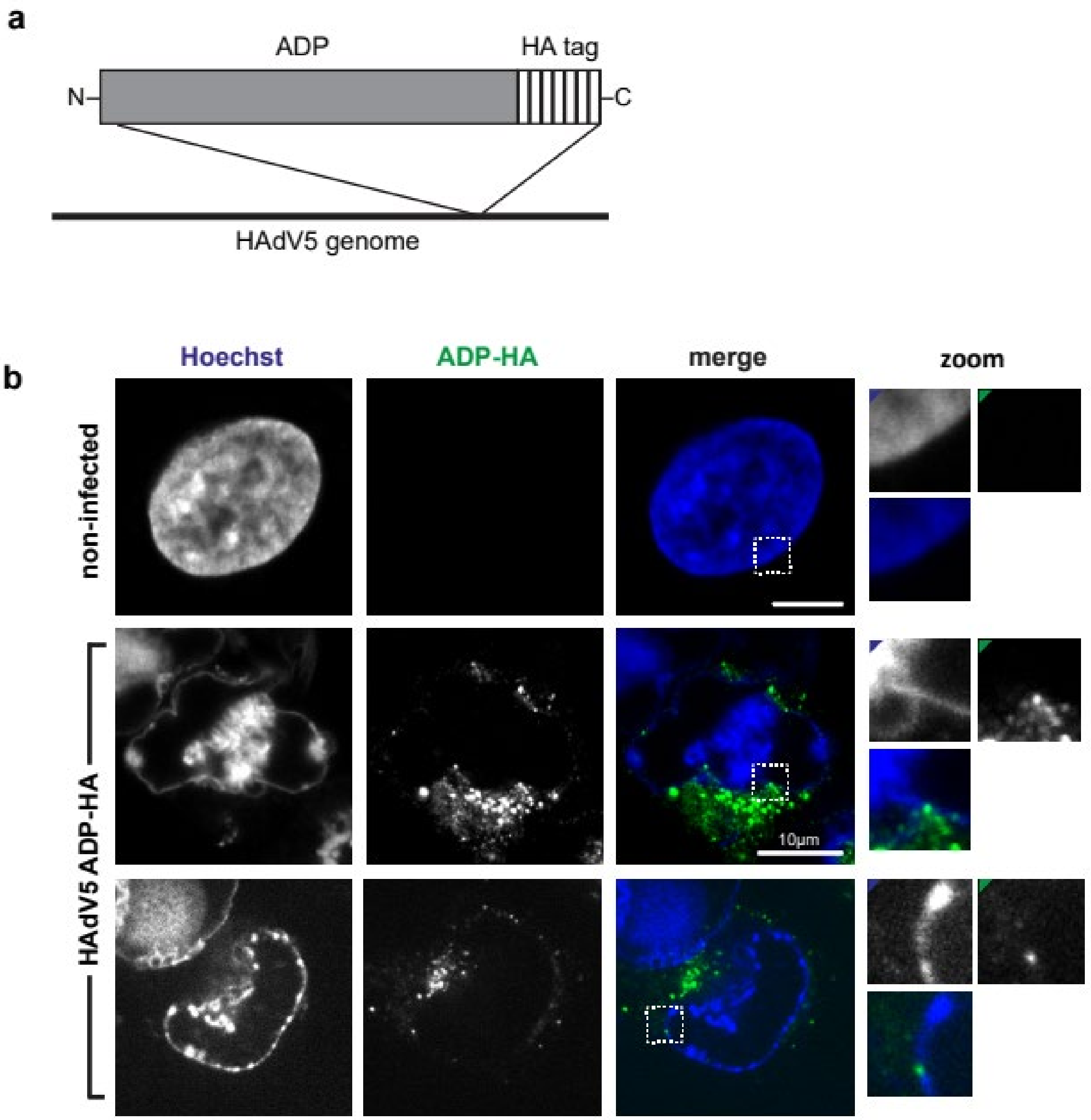

Figure S2. ADP-HA does not localize to the nuclear membrane, but to perinuclear vesicles. (**a**) Schematic of HA-labeled ADP protein in the HAdV5 genome. ADP-HA fusions were generated in the context of the HAdV5 genome by homologous recombineering in *E. coli* [16] to yield the virus strain HAdV5 ADP-HA. Mutant virus particles were generated by transfection of the linearized viral genome into H1299 cells. After transfection, the cells were analyzed for cytopathic effects. The culture supernatant was used to infect new A549 cells and progeny virions were produced by three rounds of infection (for further details see the main text). (**b**) Infection of A549 cells with HAdV5 ADP-HA. The cells were fixed at 48 hpi and prepared for immunofluorescence microscopy. The cells were stained with Hoechst 33342 (Hoechst) and the localization of ADP-HA was probed using an anti HA antibody (ADP-HA). The signal overlap is represented in color (merge). Nuclear envelope regions of interest are enlarged (zoom) with colored corners indicating the channel color. Two representative cells are shown**.**

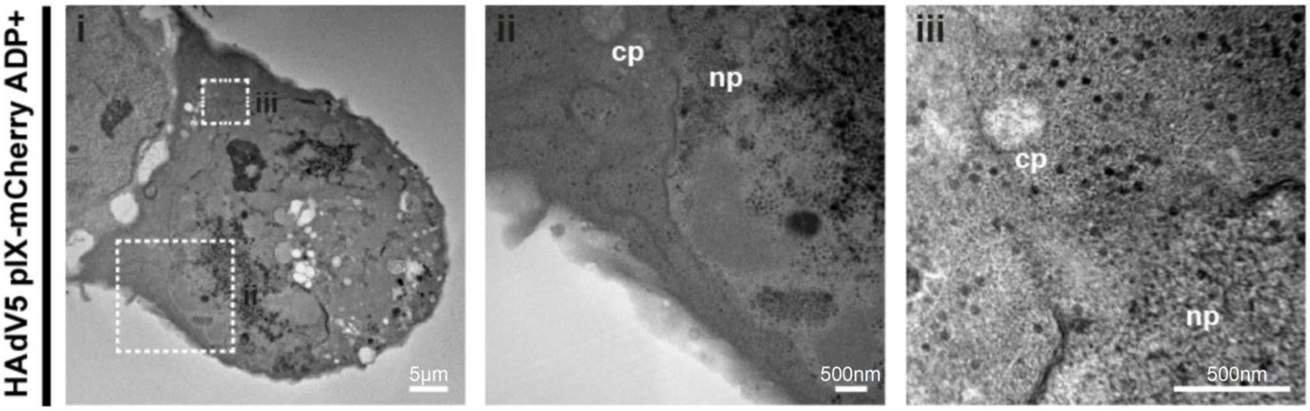

Figure S3: Site of nuclear membrane rupture and release virions into cytoplasm. An example of large-scale nuclear membrane damage in infection with HAdV5 pIX-mCherry ADP+ is shown. A549 cells were infected and prepared for classical TEM sectioning at 48 hpi. An overview of the infected cell is shown in image i), two areas of interest are shown as zooms (dashed white squares) in images ii) and iii). The cytoplasm (cp) and nucleoplasm (np) are indicated.

Supplementary methods (for Figure S3):

Cell culture dishes (µ-Dish 35 mm, high Grid-500, ibidi) were seeded with 0.5×10^5 A549 cells, infected HAdV5 pIX-mCherry and prepared at 48 hpi for TEM imaging. First, the cells were washed with PBS and fixed by incubation in 2.5 % (v/v) glutaraldehyde in PBS at 4◦C overnight. The cells were washed twice in PBS for 10 min and stained with 1 % (w/v) OsO4 in PBS for 20 min. After two wash steps in PBS and one wash step in H2O for 10 min each the cells were incubated in 1 % (w/v) uranyl acetate solution for 20 min. After two wash steps in H2O for 10 min, the cells were sequentially dehydrated in 50 %, 70 %, 90 % and 100 % (v/v) EtOH solution for 10 min each. Final dehydration in 100 % (v/v) EtOH was repeated three times before cells were embedded in epoxy resin and polymerized at 60° for 48 h. Polymerised cells of interest were removed from the cell culture dish by a 6 mm disk punch and mounted onto an epoxy resin stub. Rough trimming was performed using a scalpel to generate a flat-top pyramid with 4 sides at approximately 30° angles. 170 µm of the ibidi polymer culture dish layer on the face of the flat-top pyramid was removed by serial sectioning on an ultramicrotome (Leica Ultracut EM UCT, Leica Microsystems, Austria). The area of the pyramid block-face was further reduced by removing the access material with a scalpel. The pyramid was transferred back to the ultramicrotome and ultra-thin (□50nm) sections were cut. Carbon-coated copper grids (200 mesh, Plano) were washed in 100 % (v/v) EtOH and left to dry. The ultra-thin resin sections were collected on grids and post-contrasted with saturated uranyl acetate solution in 70 % (v/v) EtOH and imaged on a transmission electron microscope

(CM120, Philips) equipped with a LaB6 cathode operated at 80 kV and a CDD camera (MultiScan 794, Gatan, Inc.).

References:

1. Xie, Y.; Zheng, Y.; Li, H.; Luo, X.; He, Z.; Cao, S.; Shi, Y.; Zhao, Q.; Xue, Y.; Zuo, Z.; et al. GPS-Lipid: A robust tool for the prediction of multiple lipid modification sites. *Sci. Rep.* **2016**, *6*, 1–9, doi:10.1038/srep28249.
2. Drozdetskiy, A.; Cole, C.; Procter, J.; Barton, G.J. JPred4: A protein secondary structure prediction server. *Nucleic Acids Res.* **2015**, *43*, W389–W394, doi:10.1093/nar/gkv332.
3. Steentoft, C.; Vakhrushev, S.Y.; Joshi, H.J.; Kong, Y.; Vester-Christensen, M.B.; Schjoldager, K.T.B.G.; Lavrsen, K.; Dabelsteen, S.; Pedersen, N.B.; Marcos-Silva, L.; et al. Precision mapping of the human O-GalNAc glycoproteome through SimpleCell technology. *EMBO J.* **2013**, *32*, 1478–1488, doi:10.1038/emboj.2013.79.
4. Ishida, T.; Kinoshita, K. PrDOS: Prediction of disordered protein regions from amino acid sequence. *Nucleic Acids Res.* **2007**, *35*, 460–464, doi:10.1093/nar/gkm363.
5. Taherzadeh, G.; Dehzangi, A.; Golchin, M.; Zhou, Y.; Campbell, M.P. SPRINT-Gly: predicting N- and O-linked glycosylation sites of human and mouse proteins by using sequence and predicted structural properties. *Bioinformatics* **2019**, *35*, 4140–4146, doi:10.1093/bioinformatics/btz215.
6. Gautier, R.; Douguet, D.; Antonny, B.; Drin, G. HELIQUEST: A web server to screen sequences with specific α-helical properties. *Bioinformatics* **2008**, *24*, 2101–2102, doi:10.1093/bioinformatics/btn392.
7. Jumper, J.; Evans, R.; Pritzel, A.; Green, T.; Figurnov, M.; Ronneberger, O.; Tunyasuvunakool, K.; Bates, R.; Žídek, A.; Potapenko, A.; et al. Highly accurate protein structure prediction with AlphaFold. *Nature* **2021**, *596*, 583–589, doi:10.1038/s41586-021-03819-2.
8. Kelley, L.A.; Mezulis, S.; Yates, C.M.; Wass, M.N.; Sternberg, M.J.E. The Phyre2 web portal for protein modeling, prediction and analysis. *Nat. Protoc.* **2015**, *10*, 845–858, doi:10.1038/nprot.2015.053.
9. Sonnhammer, E.L.; von Heijne, G.; Krogh, A. A hidden Markov model for predicting transmembrane helices in protein sequences. *Proceedings. Int. Conf. Intell. Syst. Mol. Biol.* **1998**, *6*, 175–182.
10. Krogh, A.; Larsson, B.; Von Heijne, G.; Sonnhammer, E.L.L. Predicting transmembrane protein topology with a hidden Markov model: Application to complete genomes. *J. Mol. Biol.* **2001**, *305*, 567–580, doi:10.1006/jmbi.2000.4315.
11. Thompson, J.D.; Higgins, D.G.; Gibson, T.J. CLUSTAL W: improving the sensitivity of progressive multiple sequence alignment through sequence weighting, position-specific gap penalties and weight matrix choice. *Nucleic Acids Res*. **1994,** 22(22), 4673-4680, doi:10.1093/nar/22.22.4673
12. Needleman, S.B.; Wunsch, C.D. A general method applicable to the search for similarities in the amino acid sequence of two proteins. J. Mol. Biol. **1970**, 48(3), 443-53. doi:10.1016/0022-2836(70)90057-4 .
13. Altschul, S.F.; Madden, T.L.; Schäffer, A.A.; Zhang, J.; Zhang, Z.; Miller, W.; Lipman, D.J. Gapped BLAST and PSI-BLAST: a new generation of protein database search programs Nucleic Acids Res. **1997**, 25, 3389-3402.
14. Altschul, S.F.; Wootton, J.C.; Gertz, E.M.; Agarwala, R.; Morgulis, A.; Schäffer, A.A.; Yu, Y.K. Protein database searches using compositionally adjusted substitution matrices. FEBS J. **2005**, 272, 5101-5109.
15. Pettersen, E.F.; Goddard, T.D.; Huang, C.C.; Couch, G.S.; Greenblatt, D.M.; Meng, E.C.; Ferrin, T.E. UCSF Chimera - a visualization system for exploratory research and analysis. *J Comput Chem.* **2004**, 25(13), 1605-12.
16. Wang, H.; Bian, X.; Xia, L.; Ding, X.; Müller, R.; Zhang, Y.; Fu, J.; Stewart, A.F. Improved seamless mutagenesis by recombineering using ccdB for counterselection. *Nucleic Acids Res.* **2014**, *42*, e37, doi:10.1093/nar/gkt1339.
